# Association Between Systemic Inflammation Response Index and Specific Depressive Symptoms: Evidence From a Cross‐Sectional Survey

**DOI:** 10.1002/brb3.71541

**Published:** 2026-06-08

**Authors:** Moshui Shan, Bei Hu, Siyi Zhao, Shuhua Wang, Yu Pan

**Affiliations:** ^1^ Department of Psychiatry The 967th Hospital of the Chinese PLA Joint Logistics Support Force Dalian China; ^2^ The 93279 Unit Hospital of the Chinese PLA Dandong China; ^3^ Department of Medical Psychology The First Medical Center of Chinese PLA General Hospital Beijing China

**Keywords:** depression, inflammation, systemic inflammation response index (SIRI)

## Abstract

**Objective:**

Depression is a highly heterogeneous disorder with broad symptomatology. Systemic inflammation has been implicated in the underlying pathophysiology of depression. This study was to investigate the association between the systemic inflammation response index (SIRI) and specific depressive symptoms.

**Methods:**

Data from the National Health and Nutrition Examination Survey were employed in this cross‐sectional study. SIRI was derived from peripheral neutrophil, monocyte, and lymphocyte counts. The Patient Health Questionnaire‐9 (PHQ‐9) was administered to assess depressive symptoms. Multivariable logistic regression and generalized additive models were employed to examine the association between SIRI and specific depressive symptoms, adjusting for sociodemographic, behavioral, and medical factors.

**Results:**

Analysis of data from 30,763 participants demonstrated significant associations between SIRI and specific depressive symptoms, including anhedonia (odds ratio [OR] = 1.12, 95% confidence interval [CI]: 1.03–1.21), sleep disturbance (OR = 1.11, 95% CI: 1.04–1.19), fatigue (OR = 1.11, 95% CI: 1.04–1.19), and appetite change (OR = 1.10, 95% CI: 1.01–1.19). Nonlinear relationships were observed for anhedonia and sleep disturbance, with inflection points at SIRI = 0.67. Subgroup analyses revealed that SIRI was associated with anhedonia in adults aged 18–39 years and males, multiple depressive symptoms in Mexican American/Hispanic individuals, sleep disturbance and fatigue in adults aged 40–59 years, and fatigue in non‐Hispanic Whites.

**Conclusions:**

There is a certain degree of cross‐sectional association between SIRI and specific depressive symptoms. Future research should focus on longitudinal studies and symptom homogeneity to elucidate the underlying inflammatory mechanisms in depression.

## Graphical abstract

The study investigated the association between systemic inflammation response index (SIRI) and individual depressive symptoms. Multivariable‐adjusted associations between SIRI and symptoms of anhedonia, sleep disturbance, fatigue, and appetite changes were reported. Furthermore, nonlinear relationships were identified for anhedonia and sleep disturbance, and some heterogeneity was observed across demographic subgroups.

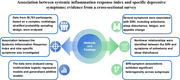

## 1 Introduction

Depression is a prevalent and heterogeneous psychiatric disorder whose symptomatology encompasses persistent sadness, anhedonia, fatigue, and cognitive impairment (Kessler and Bromet 2013; Zunszain et al. 2013). In 2019, it affected approximately 280 million people individuals globally, and the incidence rate is increasing (Malhi and Mann 2018; Vos et al. 2020). Depression is associated with substantial impairments in psychosocial functioning and a marked reduction in quality of life (Kessler and Bromet 2013; Rhee and Steffens 2020). Individuals with depression often experience social withdrawal, reduced work productivity, and poor physical health, resulting in a substantial burden on both individuals and healthcare systems (McCarron et al. 2021).

Despite extensive research, the pathophysiology of depression remains poorly understood. Multiple theoretical frameworks have been developed to explain the pathogenesis of depression, including (e.g., serotonin, norepinephrine, and dopamine) genetic factors, environmental influences, and stress‐induced neurobiological alterations (Cui et al. 2024; Rozov et al. 2024). Notably, the increasing focus on neuroinflammation has highlighted potential pathways whereby inflammatory processes may underlie the onset of depressive symptoms. Studies have identified several inflammatory markers, including C‐reactive protein (CRP) (Chang et al. 2017; Orsolini et al. 2022), neutrophil to lymphocyte ratio (NLR) (Meng et al. 2022; Shan et al. 2022), platelet‐to‐lymphocyte ratio (PLR) (Shan et al. 2023), interleukin‐6 (IL‐6) (Hodes et al. 2016; Ting et al. 2020), and systemic immune‐inflammation index (SII) (Guo and Zhu 2024; Y. Zheng, Yin, et al. 2024), indicating an association between systemic inflammation and depression. Accumulating evidence indicates that elevated levels of these markers may affect mood regulation and neuroplasticity, supporting a central role for inflammatory processes in the pathophysiology of depression.

Recently research studies suggest that depression is related to myeloid cells and lymphocytes (Foley et al. 2022). The systemic inflammation response index (SIRI) is a novel biomarker to assess systemic inflammatory status (Qi et al. 2016). Unlike conventional inflammatory markers, SIRI provides a more integrated assessment of myeloid–lymphoid homeostasis. This composite index encompasses neutrophils and monocytes, both of which are central to innate immunity and myeloid lineage activity, together with lymphocytes, which govern adaptive immune responses. As such, SIRI serves as a reflection of the bidirectional communication between peripheral inflammation and immune dynamics within the central nervous system. It has been applied in various medical fields to evaluate inflammatory status and predict patient outcomes in diverse conditions (Cai et al. 2023; Cao et al. 2023; Huang et al. 2024). Previous research has suggested that SIRI may reflect the levels of systemic inflammation and its potential impact on mental health, including depressive symptoms (Ma et al. 2024; Ninla‐Aesong et al. 2024). Nonetheless, the evidence regarding the link between SIRI and depression remains sparse and necessitates further work within the realm of psychiatric pathophysiology.

To date, most studies investigating the depression have focused on the diagnostic categorization, wherein participants are dichotomized by clinical criteria. Although these approaches have yielded valuable insights, they are limited in elucidating the correlation between specific depressive symptoms and these biomarkers. Depression is highly heterogeneous. There is increasing recognition of the importance of symptom‐level analysis for a comprehensive understanding of the pathophysiology of depression and the development of targeted interventions.

This study aimed to investigate the association between specific depressive symptoms and SIRI, thereby enhancing the comprehension of the complex interplay between systemic inflammation and the subtleties of depressive symptoms in patients. Through examining these associations, this study may contribute valuable insights for the development of personalized intervention strategies.

## 2 Methods

### 2.1 Study Population

Data comprising this analysis were obtained from the National Health and Nutrition Examination Survey (NHANES) database. NHANES is a nationally representative program administered by the National Center for Health Statistics (NCHS). The survey is centered on a multistage, stratified probability sampling design, constructed to yield a sample that is representative of the civilian, non‐institutionalized population (Zipf et al. 2013). The study employs a modular approach to data acquisition, integrating information from three primary sources: interviews, physical examinations, and laboratory testing. Blood collection and Patient Health Questionnaire‐9 (PHQ‐9) administrations were conducted simultaneously at the NHANES mobile examination centers (MEC). Prior to conducting any laboratory work, staff completed rigorous training in standardized protocols to ensure consistency. Participants completed the questionnaire at the MEC via a computer‐assisted personal interviewing (CAPI). The NHANES datasets are adjusted for nonresponse, oversampling, and the complex survey design, by applying appropriate weights to facilitate analyses that reflect national estimates. Data are publicly available and can be accessed via the NHANES website. Relevant cycles were selected on the basis of the availability of depression screening tools and complete blood count (CBC) results. Detailed documentation on the NHANES website provides information on the methodologies used in data collection and participant recruitment. Seven cycles of survey data (2005–2018) were analyzed in this study.

Eligibility for inclusion was defined as follows: (1) individuals with an age of 18 years or above; (2) complete data for both CBC and depressive symptom screening using the PHQ‐9. Exclusion criteria were (1) history of cancer or malignancies; (2) use of medications that could potentially affect blood cell concentrations (e.g., nonsteroidal anti‐inflammatory drug, corticosteroids, and anti‐infective agents).

The NCHS Ethics Review Board approved all data collection methods, and the NHANES database adhered to strict ethical standards (Protocol #2005‐06, Protocol #2011‐17) (https://www.cdc.gov/nchs/nhanes/irba98.htm). As a standard procedure, written informed consent was acquired from all individuals preceding their inclusion in the study. Given the retrospective nature of the analysis, which uses publicly available, de‐identified data, ethical review, and informed consent were waived for this study.

### 2.2 Systemic Inflammation Response Index

According to previous population‐based studies focusing on systemic inflammation (You, Ablitip, et al. 2024; You, Li, et al. 2024), SIRI was computed using the CBC parameters. The index was determined by the following standard formula: (neutrophil count × monocyte count)/lymphocyte count. Neutrophil, monocyte, and lymphocyte counts were measured on the basis of the five‐part differential. The comprehensive analytical protocols were outlined in the official NHANES guidelines.

### 2.3 Depressive Symptoms Assessment

The PHQ‐9, a widely validated instrument for screening the depressive symptoms, was administered to assess depressive symptoms. The PHQ‐9 comprises nine items, each corresponding to a specific symptom of depression, with participants rating the frequency of these symptoms over the past 2 weeks on a scale from 0 (not at all) to 3 (nearly every day). Each item description of PHQ‐9 is presented in Table S1. It has demonstrated strong validity and reliability, effectively distinguishing between varying levels of depressive symptoms across diverse populations (Kroenke et al. 2001). According to the literature, a symptom was considered present if the response to that item was “more than half the days” or “nearly every day” (≥2) (Jokela et al. 2016).

### 2.4 Covariates

The selection of confounding variables for adjustment was informed by prior literature (You, Zheng, et al. 2025; You, Li, et al. 2025), theoretical rationale, data availability, and clinical practice to minimize confounding. These covariates included sociodemographic factors, health behaviors, and existing medical conditions, all of which have been shown to independently influence mental health outcomes. Specifically, these covariates encompassed demographic, socioeconomic, behavioral, and clinical factors, including age, sex, race/ethnicity, education, marital status, poverty‐income ratio, body mass index (BMI), comorbidities, excessive alcohol use, smoking status, and antidepressant medication use. Participants were stratified into four groups: Mexican American/other Hispanic, non‐Hispanic White, non‐Hispanic Black, and other races. Educational level was classified as follows: below high school, high school, and above high school. Marital status was classified as married/living with a partner, never married, and divorced/separated/widowed. These categories reflect different levels of social support and relationship stability, which may influence mental health. The poverty‐income ratio was included to capture socioeconomic status and its potential impact on mental health. BMI was calculated using the formula: weight (kg)/height (m)^2, which is a standard measure of body size and a well‐established correlate of various health conditions, including mental health disorders. BMI was categorized according to the standard WHO definitions (Weir and Jan 2025). Smoking status was classified as never, former, or current smoker. Never smokers were defined as those with a lifetime consumption of fewer than 100 cigarettes. Former smokers were defined as individuals with a lifetime consumption of over 100 cigarettes who had subsequently quit. Current smokers were identified as those reporting current use of cigarettes. Alcohol use was based on self‐reported frequency and amount of alcohol consumption. Excessive alcohol use was defined as an average daily intake of five or more alcoholic drinks over the preceding year (Meng et al. 2022). Medical conditions included a range of chronic health issues: asthma, anemia, arthritis, liver conditions, thyroid problems, cardiovascular disease, hypertension, diabetes, and dyslipidemia (Table S2). By controlling for these covariates, we aimed to minimize bias and enhance the validity and reliability of the findings regarding the relationship between SIRI and depressive symptoms.

### 2.5 Statistical Methods

To incorporate the complex survey design and produce nationally representative estimates, we utilized the 2‐year full sample examination weights (WTMEC2YR) in accordance with the NHANES analytic guidelines. For analyses combining multiple survey cycles, weights were adjusted by dividing by 7. Stratification, primary sampling units, and the adjusted weights were incorporated into weighted logistic regression models to account for the complex survey designs. Outlier values were handled using the winsorization, which operates by recoding extreme observations to the nearest values within an acceptable range. Specifically, for continuous variables with outliers, we winsorized the data at values exceeding three standard deviations (Riis et al. 2015). The main analyses used the winsorized SIRI values as the exposure variable. This approach mitigates the influence of outliers on the overall results and maintains the robustness of the analytical findings. Continuous variables were summarized using means and standard errors (SEs) or medians and interquartile ranges (IQR), depending on their distribution. Categorical variables were presented as frequencies and percentages. Multivariable logistic regression models were used to investigate the association between SIRI and depressive symptoms. In the crude model, no variables were included. Model I was adjusted for basic sociodemographic variables. Model II was further controlled for medical and behavioral factors. By adjusting for these covariates, we aimed to assess the independent relationship of SIRI on depressive symptoms. To examine the potential nonlinear relationship between SIRI and depressive symptoms, we fitted generalized additive models (GAM). The likelihood ratio test was used to compare the piecewise linear model with the simple linear model. Given the sample size and research objective, the two‐piecewise linear model was employed to capture the threshold effect while avoiding the overfitting associated with more flexible nonlinear specifications. To explore potential variations in the SIRI‐depressive symptoms association, subgroup analyses were performed across different demographic strata (age, sex, and race). To evaluate for heterogeneity of effects across subgroups, interaction terms were incorporated into the regression models.

The statistical analyses were performed with R software package (version 4.4.2, http://www.r‐project.org). The mgcv and survey packages in R were used for multivariable regression and GAM analyses (e.g., survey::svyglm, mgcv::gam). Statistical significance was defined as a two‐tailed *p* value below 0.05.

## 3 Results

### 3.1 Baseline Characteristics of Populations

**FIGURE 1:**
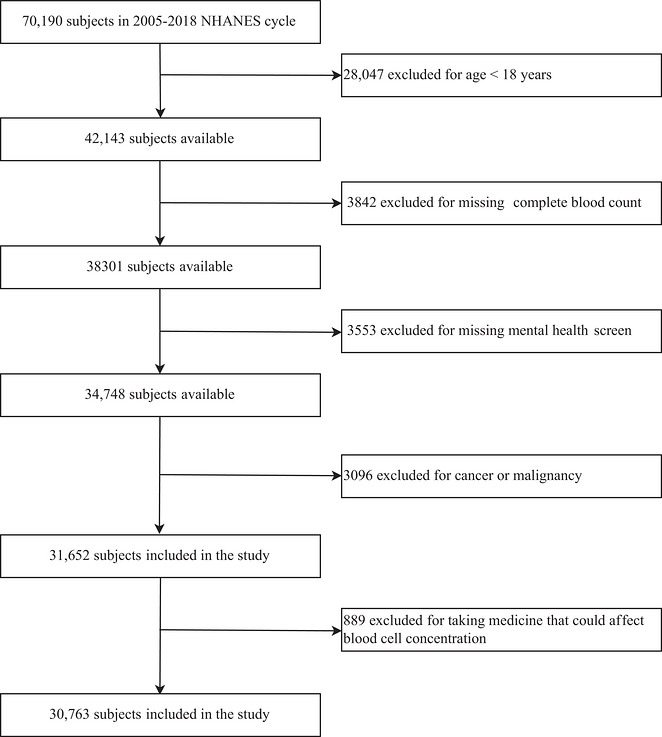
Flow chart of study. NHANES, Nutrition Examination Survey.

The NHANES dataset from 2005 to 2018 included 70,190 participants. Of these, 28,047 participants were excluded due to being under 18 years of age. Additionally, 3842 participants were excluded due to missing blood count data, 3553 due to missing mental health screening results, 3096 due to a history of cancer or malignancy, and 889 due to the use of medications known to affect blood cell concentrations. The final analytic sample included 30,763 participants. Figure 1 presents the flowchart for participant selection. The median age of the participants was 44 years (IQR: 30–57). Of the participants, 15,116 (49.28%) were male, 8367 (15.17%) were Mexican American/other Hispanic, 12,203 (65.89%) were non‐Hispanic White, 6767 (11.38%) were non‐Hispanic Black, and 3426 (7.56%) were other races. Table 1 presents the weighted baseline characteristics of the participants.

**TABLE 1:** Weighted characteristics of subjects in Nutrition Examination Survey (NHANES) from 2005 to 2018.

Characteristic	Overall (*n* = 30763)	SIRI, *n* (%)^a^					
		*Q*1: <0.67 (*n *= 7540)	*Q*2: ≥0.68–0.99 (*n *= 7550)	*Q*3: ≥1.00–1.46 (*n *= 7865)	*Q*4: ≥1.47 (*n* = 7808)	*p* value	
Age, median (IQR), years	44.00 (30.00, 57.00)	42.00 (29.00, 55.00)	42.00 (30.00, 55.00)	44.00 (31.00, 57.00)	46.00 (32.00, 60.00)		<0.01
18 to ≤39	12,140 (40.10)	3112 (44.05)	3049 (42.09)	3033 (38.61)	2946 (36.59)		<0.01
40 to ≤59	10,002 (37.67)	2571 (37.98)	2540 (38.10)	2644 (39.19)	2247 (35.48)		
60 to ≤79	7248 (19.16)	1699 (16.42)	1722 (17.74)	1833 (19.23)	1994 (22.62)		
≥80	1373 (3.06)	158 (1.55)	239 (2.07)	355 (2.97)	621 (5.31)		
Sex							0.03
Male	15,116 (49.28)	3319 (43.69)	3575 (47.65)	3992 (50.47)	4230 (54.06)		
Female	15,647 (50.72)	4221 (56.31)	3975 (52.35)	3873 (49.53)	3578 (45.94)		
Race/Ethnicity							<0.01
Mexican American/Other Hispanic	8367 (15.17)	1784 (15.27)	2235 (16.30)	2312 (15.37)	2036 (13.81)		
Non‐Hispanic White	12,203 (65.89)	1823 (51.32)	2893 (65.46)	3469 (69.81)	4018 (73.93)		
Non‐Hispanic Black	6767 (11.38)	2878(22.98)	1550 (10.50)	1253 (7.74)	1086 (6.69)		
Other races	3426 (7.56)	1055 (10.43)	872 (7.74)	831 (7.08)	668 (5.58)		
Education							<0.01
Below high school	6983 (15.67)	1601 (15.56)	1729 (14.83)	1842 (15.60)	1811 (16.64)		
High school	6635 (23.47)	1508 (21.42)	1588 (22.75)	1690 (23.19)	1849 (26.05)		
Above high school	15,166 (60.86)	3853 (63.02)	3778 (62.42)	3846 (61.22)	3689 (57.31)		
Marital status							<0.01
Married/Living with partner	17,319 (63.38)	4087 (62.52)	4378 (65.41)	4541 (64.79)	4313 (60.70)		
Never married	5989 (19.55)	1650 (22.01)	1395 (19.36)	1467 (18.23)	1477 (19.13)		
Divorced/Separated/Widowed	5953 (17.07)	1351 (15.47)	1409 (15.23)	1500 (16.98)	1693 (20.16)		
Poverty income ratio							<0.01
<1 (lowest income)	6296 (14.69)	1563 (15.56)	1477 (13.99)	1613 (14.09)	1643 (15.28)		
1 to 2	7485 (20.30)	1692 (19.53)	1803 (19.65)	1927 (19.58)	2063 (22.28)		
2 to 4	7392 (28.85)	1855 (29.50)	1812 (28.53)	1878 (29.07)	1847 (28.43)		
≥4 (highest income)	7037 (36.16)	1771 (35.41)	1819 (37.84)	1780 (36.26)	1667 (34.02)		
BMI (kg/m^2^**)							<0.01
<18.5	537 (1.69)	177 (2.33)	117 (1.52)	113 (1.58)	130 (1.45)		
18.5 to <25	8704 (29.11)	2454 (34.98)	2216 (30.20)	2063 (26.26)	1858 (25.32)		
25 to 30	9863 (32.49)	2455 (33.46)	2477 (32.95)	2506 (32.28)	2425 (31.50)		
>30	11,383 (36.71)	2411 (29.24)	2694 (34.33)	3123 (39.88)	3155 (41.73)		
Medical conditions	1.27 (0.01)	1.08 (0.02)	1.15 (0.02)	1.28 (0.02)	1.52 (0.02)		<0.01
Excessive alcohol use							<0.01
Yes	4164 (15.59)	806 (12.37)	936 (14.32)	1119 (16.14)	1303 (18.66)		
No	21,189 (84.41)	5244 (87.63)	5271 (85.68)	5398 (83.86)	5276 (81.34)		
Smoking status							<0.01
Never	16,853 (56.61)	4575 (63.88)	4330 (58.81)	4257 (56.26)	3691 (49.17)		
Former	6507 (22.95)	1356 (19.99)	1573 (23.04)	1687 (22.96)	1891 (25.19)		
Current	6147 (20.44)	1227 (16.13)	1372 (18.15)	1620 (20.78)	1928 (25.64)		
Antidepressant							<0.01
Yes	2888 (11.81)	570 (10.27)	665 (10.77)	773 (12.38)	880 (13.43)		
No	27,875 (88.19)	6970 (89.73)	6885 (89.23)	7092 (87.62)	6928 (86.57)		
SIRI, mean (SE)	1.22 (0.01)	0.50 (0.002)	0.83 (0.001)	1.20 (0.002)	2.18 (0.009)		<0.01

Abbreviations: BMI, body mass index; IQR, interquartile range; SE, standard error; SIRI, systemic inflammation response index.

^a^ Data from NHANES (2005–2018 cycles), are demonstrated as unweighted number of participants and weighted percentages, or weighted mean and weighted standard error, weighted to be nationally representative.

^**^ p value <0.05

### 3.2 Association Between SIRI and Specific Depressive Symptoms

Table 2 shows the correlations between SIRI and depressive symptoms, with the latter treated as categorical variables. The analysis revealed significant correlations between SIRI and several depressive symptoms, including anhedonia (odds ratio [OR] = 1.18, 95% confidence interval [CI]: 1.10–1.25), depressed mood (OR = 1.17, 95% CI: 1.08–1.26), sleep disturbance (OR = 1.21, 95% CI: 1.15–1.28), fatigue (OR = 1.18, 95% CI: 1.12–1.24), appetite change (OR = 1.13, 95% CI: 1.06–1.21), low self‐esteem (OR = 1.14, 95% CI: 1.05–1.24), and concentration problems (OR = 1.12, 95% CI: 1.04–1.20). After controlling for demographic and socioeconomic covariates (including age, sex, race, education, marital status, and poverty‐income ratio), the associations with SIRI remained significant in Model I. After full adjustment, SIRI remained independently associated with anhedonia (OR = 1.12, 95% CI: 1.03–1.21), sleep disturbance (OR = 1.11, 95% CI: 1.04–1.19), fatigue (OR = 1.11, 95% CI: 1.04–1.19), and appetite change (OR = 1.10, 95% CI: 1.01–1.19).

**TABLE 2:** Weighted multivariable logistic regression analyses for systemic inflammation response index (SIRI) and specific depressive symptom.

Depressive symptom	Crude model		Adjust model I		Adjust model II	
	OR (95% CI)	*p* value	OR (95% CI)	*p* value	OR (95% CI)	*p* value
Anhedonia	1.18 (1.10, 1.25)	<0.001	1.17 (1.09, 1.25)	<0.001	1.12 (1.03, 1.21)	0.007
Depressed mood	1.17 (1.08, 1.26)	<0.001	1.14 (1.05, 1.24)	0.002	1.07 (0.97, 1.18)	0.17
Sleep disturbance	1.21 (1.15, 1.28)	<0.001	1.20 (1.13, 1.27)	<0.001	1.11 (1.04, 1.19)	0.002
Fatigue	1.18 (1.12, 1.24)	<0.001	1.19 (1.12, 1.25)	<0.001	1.11 (1.04, 1.19)	0.004
Appetite change	1.13 (1.06, 1.21)	<0.001	1.18 (1.10, 1.26)	<0.001	1.10 (1.01, 1.19)	0.02
Low self‐esteem	1.14 (1.05, 1.24)	0.003	1.12 (1.01, 1.23)	0.03	1.03 (0.92, 1.15)	0.62
Concentration problems	1.12 (1.04, 1.20)	0.004	1.12 (1.04, 1.22)	0.006	1.06 (0.97, 1.16)	0.23
Psychomotor changes	1.10 (0.99, 1.23)	0.09	1.07 (0.95, 1.22)	0.26	1.01 (0.88, 1.16)	0.88
Suicidal ideation	1.04 (0.86, 1.25)	0.72	0.96 (0.79, 1.18)	0.73	0.95 (0.76, 1.18)	0.62

*Note*: Crude model: unadjusted. Adjust model I: adjusted for age, sex, race, education level, marital status, and poverty income ratio. Adjust model II: adjusted for age, sex, race, education level, marital status, poverty income ratio, body mass index, medical conditions, smoking status, alcohol use, and antidepressant use.

Abbreviations: CI, confidence interval; OR, odds ratio.

### 3.3 Nonlinear Relationship Between SIRI and Depressive Symptoms Severity

**FIGURE 2:**
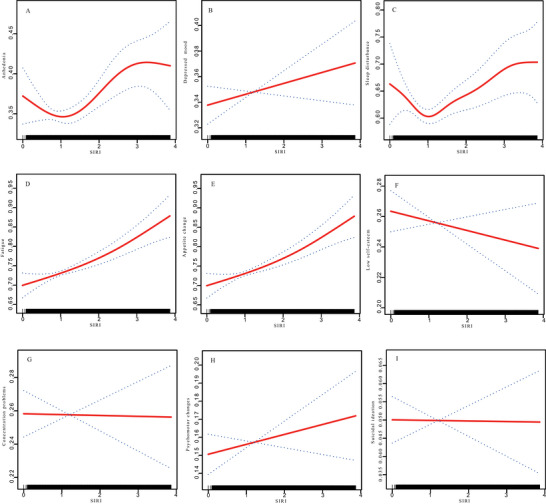
The nonlinear relationship between SIRI and depressive symptoms by smooth curve fitting. (A) The nonlinear relationship between SIRI and anhedonia. (B) The nonlinear relationship between SIRI and depressed mood. (C) The nonlinear relationship between SIRI and sleep disturbance. (D) The nonlinear relationship between SIRI and fatigue. (E) The nonlinear relationship between SIRI and appetite change. (F) The nonlinear relationship between SIRI and low self‐esteem. (G) The nonlinear relationship between SIRI and concentration problems. (H) The nonlinear relationship between SIRI and psychomotor changes. (I) The nonlinear relationship between SIRI and suicidal ideation.

Nonlinear relationships between SIRI and specific depressive symptoms, namely anhedonia and sleep disturbance, were observed after adjusting for all covariates (Figure 2A–I). A two‐piecewise linear regression model was employed to identify the inflection point, which was determined to be 0.67 (Table 3). For anhedonia, a negative association with SIRI was observed below the inflection point (*β* = −0.11, 95% CI: −0.22 to −0.01). In contrast, a positive association was observed above the inflection point (*β* = 0.03, 95% CI: 0.01–0.04). For sleep disturbance, a negative association with SIRI was observed below the inflection point (*β* = −0.16, 95% CI: −0.29 to −0.03). Similarly, a positive association was observed above the inflection point (*β* = 0.03, 95% CI: 0.01–0.05).

**TABLE 3:** Threshold effect analysis of systemic inflammation response index (SIRI) on severity of depression.

Outcome	Anhedonia		Sleep disturbance	
	*β* (95% CI)	*p* value	*β* (95% CI)	*p* value
Fitting model by two‐piecewise linear regression				
<0.67	−0.11 (−0.22, −0.01)	0.03	−0.16 (−0.29, −0.03)	0.02
≥0.67	0.03 (0.01, 0.04)	<0.001	0.03 (0.01, 0.05)	<0.001
*P* for log likelihood ratio test	0.009		0.007	

*Note*: Adjusted for age, sex, race, education level, marital status, poverty income ratio, body mass index, medical conditions, smoking status, alcohol use, and antidepressant use.

Abbreviations: CI, confidence interval; OR, odds ratio.

### 3.4 Subgroups Analyses

**FIGURE 3:**
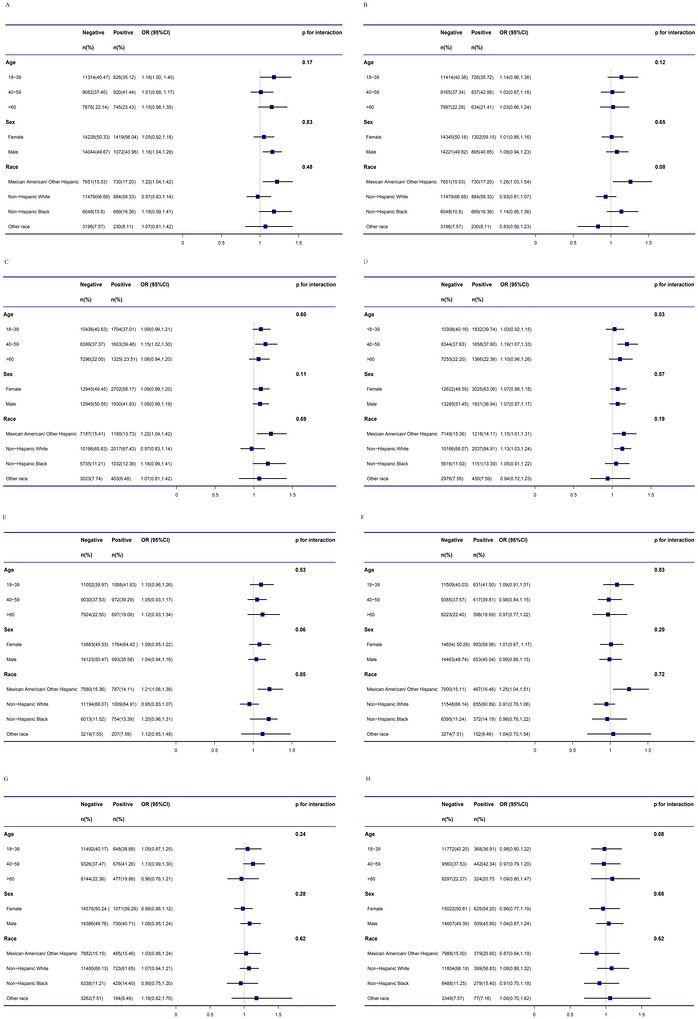
Subgroup analysis for SIRI and specific depressive symptoms. (A) Subgroup analysis between SIRI and anhedonia. (B) Subgroup analysis between SIRI and depressed mood. (C) Subgroup analysis between SIRI and sleep disturbance. (D) Subgroup analysis between SIRI and fatigue. (E) Subgroup analysis between SIRI and appetite change. (F) Subgroup analysis between SIRI and low self‐esteem. (G) Subgroup analysis between SIRI and concentration problems. (H) Subgroup analysis between SIRI and psychomotor changes. OR, odds ratio.

The stratified analyses revealed distinct association patterns between SIRI and various depressive symptoms across demographic subgroups (Figure 3A–H). Among young adults (18–39 years), elevated SIRI levels showed significant associations with anhedonia symptoms (OR = 1.18, 95% CI: 1.00−1.40), with similar effects observed in male participants (OR = 1.16, 95% CI: 1.04–1.28).

Notably, Mexican American/Other Hispanic individuals demonstrated the most consistent associations across multiple symptom domains. This population exhibited significant associations between SIRI and several depressive symptoms: anhedonia (OR = 1.22, 95% CI: 1.04–1.42), depressed mood (OR = 1.26, 95% CI: 1.03–1.54), sleep disturbance (OR = 1.22, 95% CI: 1.04–1.42), appetite changes (OR = 1.21, 95% CI: 1.06–1.38), and low self‐esteem (OR = 1.25, 95% CI: 1.04–1.51).

In middle‐aged adults (40–59 years), elevated SIRI levels were associated with sleep disturbance (OR = 1.15, 95% CI: 1.02–1.30) and fatigue (OR = 1.19, 95% CI: 1.07–1.33). Significant associations were also observed between SIRI and fatigue in non‐Hispanic White individuals (OR = 1.13, 95% CI: 1.03–1.24).

No significant association was observed between SIRI and concentration problems or psychomotor changes. However, a significant interaction between appetite changes and SIRI was observed across different age groups (OR = 1.03 [95% CI: 0.92–1.15] vs. 1.19 [1.07–1.33] vs. 1.10 [0.96–1.26], *p* for interaction = 0.03). Subgroup analysis between SIRI and suicidal ideation was not conducted due to insufficient positive sample size (*n *= 327, 0.01%).

### 3.5 Sensitivity Analyses

A sensitivity analysis was conducted, treating each PHQ‐9 item score as a continuous variable, to assess the stability of the results. Table 4 presents the correlations between SIRI and the severity of various depressive symptoms. In the crude model, SIRI was associated with anhedonia (*β* = 0.04, 95% CI: 0.02–0.05), depressed mood (*β* = 0.03, 95% CI: 0.02–0.05), sleep disturbance (*β* = 0.07, 95% CI: 0.05–0.09), fatigue (*β* = 0.06, 95% CI: 0.04–0.08), appetite change (*β* = 0.03, 95% CI: 0.02–0.05), low self‐esteem (*β* = 0.02, 95% CI: 0.01–0.03), and psychomotor changes (*β* = 0.01, 95% CI: 0.003–0.02). Similar trends were observed in Model I. After full adjustment, SIRI remained significantly associated with anhedonia (*β* = 0.02, 95% CI: 0.007–0.04), sleep disturbance (*β* = 0.03, 95% CI: 0.01–0.05), fatigue (*β* = 0.03, 95% CI: 0.01–0.06), and appetite change (*β* = 0.02, 95% CI: 0.002–0.04).

**TABLE 4:** Weighted multivariable logistic regression analyses for systemic inflammation response index (SIRI) and specific symptom severity.

Depressive symptom	Crude model		Adjust model I		Adjust model II	
	*β* (95% CI)	*p* value	*β* (95% CI)	*p* value	*β* (95% CI)	*p* value
Anhedonia	0.04 (0.02, 0.05)	<0.001	0.04 (0.02, 0.05)	<0.001	0.02 (0.007, 0.04)	0.007
Depressed mood	0.03 (0.02, 0.05)	<0.001	0.03 (0.02, 0.04)	<0.001	0.01 (−0.001, 0.03)	0.07
Sleep disturbance	0.07 (0.05, 0.09)	<0.001	0.06 (0.04, 0.08)	<0.001	0.03 (0.01, 0.05)	0.008
Fatigue	0.06 (0.04, 0.08)	<0.001	0.07 (0.05, 0.09)	<0.001	0.03 (0.01, 0.06)	0.002
Appetite change	0.03 (0.02, 0.05)	<0.001	0.04 (0.03, 0.06)	<0.001	0.02 (0.002, 0.04)	0.03
Low self‐esteem	0.02 (0.01, 0.03)	0.002	0.02 (0.001, 0.03)	0.03	−0.001 (−0.01, 0.01)	0.96
Concentration problems	0.01 (−0.01, 0.03)	0.05	0.01 (−0.001, 0.03)	0.06	0.002 (−0.01, 0.02)	0.78
Psychomotor changes	0.01 (0.003, 0.02)	0.008	0.01 (0.001, 0.02)	0.04	0.003 (−0.01, 0.01)	0.64
Suicidal ideation	0.005 (−0.001, 0.01)	0.065	0.002 (−0.003, 0.007)	0.38	−0.001 (−0.001, 0.005)	0.96

*Note*: Crude model: unadjusted. Adjust model I: adjusted for age, sex, race, education level, marital status, and poverty income ratio. Adjust model II: adjusted for age, sex, race, education level, marital status, poverty income ratio, body mass index, medical conditions, smoking status, alcohol use, and antidepressant use.

Abbreviation: CI, confidence interval.

Given the potential confounding effect of comorbid health conditions, sensitivity analyses were performed by sequentially excluding each chronic disease group. The observed associations remained consistent across all sensitivity analyses. The results are listed in Tables S3–S11.

## 4 Discussion

The relationship between inflammatory factors and depressive symptoms has become a focus of research in recent years, with a mounting body of evidence supporting the notion that inflammation plays a crucial role in the pathophysiology of depression (Bertollo et al. 2025; Chen et al. 2025; Herzog et al. 2025). Numerous studies have documented elevated levels of pro‐inflammatory cytokines, such as IL‐6, CRP, and tumor necrosis factor‐alpha (TNF‐α), in individuals with depression. These findings suggest that an inflammatory response may influence neurotransmitter systems (Cheng et al. 2022), neuroplasticity (Fuchs et al. 2004; Teyler and Cavus 2007), and brain circuitry involved in mood regulation (Artigas 2021; W. Zheng, Zhang, et al. 2024), thereby contributing to depressive symptoms. From a mechanistic perspective, multiple pathways have been posited to account for the link between inflammation and depression. One prominent hypothesis is the influence of pro‐inflammatory cytokines on neurotransmitter systems, particularly the monoamine hypothesis, which posits that alterations in serotonin and dopamine levels may result from cytokine‐mediated activation of indoleamine 2,3‐dioxygenase (IDO), leading to a reduction in tryptophan availability (Bertollo et al. 2025; Tiwari and Paramanik 2025). Furthermore, inflammatory factors may modulate the signaling of brain‐derived neurotrophic factor (BDNF), which is critical for neurogenesis and synaptic plasticity in the hippocampus (Fan et al. 2025; Hu et al. 2025). The chronic release of pro‐inflammatory cytokines can lead to an increased activation of the hypothalamic–pituitary–adrenal (HPA) axis, resulting in heightened levels of cortisol, a stress hormone that has been shown to have adverse effects on mental health (Gelman et al. 2015). Despite substantial evidence linking inflammatory processes to depressive symptoms, challenges persist in this field. The primary challenge is determining whether increased inflammation is a universal feature of depression or characteristic of specific subgroups. Additionally, the heterogeneity of depression complicates the elucidation of its underlying mechanisms. Depressive symptoms exhibit considerable variability in manifestation among individuals. Consequently, establishing a consistent inflammatory profile for depression is challenging and has yielded inconsistent results across studies.

SIRI constitutes a novel hematologic biomarker distinguished by its unique cellular composition. Compared with other biomarkers like SII, SIRI specifically emphasizes myeloid lineage cells, providing a more nuanced representation of systemic inflammatory dynamics. This mechanistic specificity holds particular relevance for the immuno‐inflammatory hypothesis of depression, which posits that chronic low‐grade inflammation contributes to the pathogenesis of depression. This myeloid‐focused profile enhances the biological plausibility for psychiatric research, given emerging evidence of microglial activation (tissue‐resident macrophages of myeloid origin) and blood–brain barrier permeability increases. The complexity of inflammatory regulation is reflected in the fact that both high and low SIRI levels can indicate inflammatory processes. These levels may reflect imbalances between pro‐inflammatory and anti‐inflammatory cytokines, or the activation of inflammatory pathways, such as the NF‐κB pathway (de Deus et al. 2025). This study determined the independent association of SIRI with depressive symptoms and the potential nonlinear relationship. The results revealed an association between inflammation and neurovegetative symptoms, including sleep problems, fatigue, and appetite changes, consistent with prior research (Jokela et al. 2016). Moreover, the nonlinear relationship between SIRI and anhedonia or sleep disturbance (inflection point at 0.67) aligns with emerging evidence linking inflammatory indices to depression risk (Zhu et al. 2025). When SIRI < 0.67, a one‐unit increase in SIRI is associated with a 0.11 reduction in anhedonia score or a 0.16 reduction in sleep disturbance score. When SIRI ≥ 0.67, each additional SIRI unit is associated with a 0.03 increase in anhedonia score or sleep disturbance score. This suggests that low systemic inflammation may be neuroprotective or compensatory, whereas higher levels reflect pathological overactivation.

The results of the study, particularly regarding fatigue, sleep, appetite, and anhedonia, align with the concept of immune‐metabolic depression (Penninx et al. 2025). Immune‐metabolic depression is a subtype characterized by atypical, energy‐related symptoms, coupled with systemic inflammation, abdominal obesity, dyslipidemia, insulin/leptin resistance, and mitochondrial dysfunction. This concept posits bidirectional interactions between inflammatory dysregulation, metabolic dysfunction, and neuropsychiatric symptoms. Given its integration of neutrophils, monocytes, and lymphocytes, SIRI reflects systemic immune activation. Its specific cellular composition, particularly the emphasis on myeloid cells (neutrophils, monocytes), may also make it a relevant biomarker for investigating the immuno‐metabolic crosstalk implicated in this depression subtype, where inflammation and metabolism are intertwined. Although this study demonstrates associations between SIRI and specific symptom domains, including novel nonlinear relationships, future research exploring interventions targeting dysregulated immune‐metabolic pathways is warranted to determine if modulating these pathways can effectively alleviate these specific depressive symptoms.

The results indicate that suicidal ideation and psychomotor changes were not significantly associated with SIRI. Potential explanations for these findings include methodological limitations, specific sample characteristics, cultural or contextual factors (such as stigma), and the heterogeneous nature of inflammation. Therefore, SIRI should not be used in isolation to comprehensively assess the risk of suicide or psychomotor issues among individuals with depression.

To date, most research has focused on categorical diagnoses of depression, overlooking symptom‐level heterogeneity. This approach inherently overlooked the substantial heterogeneity of depressive symptoms and the variability among individuals experiencing MDD. Classifying depression solely based on diagnostic criteria may obscure the interplay between inflammation and the pathophysiology of symptoms. Future research should prioritize elucidating the mechanisms of symptom heterogeneity to advance our understanding of the underlying psychopathology of depression.

This study had several advantages compared to previous works. First, due to the large size of the sample and the complex sampling method used, this study is considered to represent a good population. Second, this study took into account multiple confounding factors. Third, this study examines the relationship between depressive symptoms and novel inflammatory factors at the level of depressive symptoms, providing a new perspective on the mechanism of depression. Meanwhile, there were several limitations in the study. First, due to the cross‐sectional design of the NHANES survey, causal inference is not possible. Reverse causation is a plausible alternative explanation. For example, depression is often accompanied by sleep disturbances, which can directly activate the innate immune system and promote the release of inflammatory markers. Thus, although an association was observed, the temporal direction remains entirely unclear. Second, CBC was measured only once, which may introduce bias. A more accurate assessment of the subject's inflammatory status could be achieved through repeated blood cell counts. Third, residual confounding from unmeasured variables persists. Unmeasured or inadequately measured variables may bias our estimates, such as acute/subclinical infections, detailed dietary patterns, and psychosocial stress. Fourth, the extensive subgroup analyses were conducted without adjustment for multiple comparisons, which may have inflated the Type I error rate. Consequently, these subgroup findings should be considered hypothesis‐generating and exploratory, warranting replication in independent, well‐powered cohorts. Additionally, the small sample sizes limited the interpretability of both the findings for the other race subgroup and the analysis of suicidal ideation. Finally, the utility of SIRI in detecting subtle, chronic low‐grade inflammatory changes associated with depression remains uncertain, potentially limiting its validity in this context.

## 5 Conclusion

In summary, there is a certain degree of cross‐sectional correlation between inflammation and specific symptoms of depression, including anhedonia, sleep problems, fatigue, and appetite changes. Moreover, these associations may vary across different demographic stratifications. In future, depression management has the potential to evolve from a uniform, one‐size‐fits‐all paradigm to personalized strategies firmly grounded in the pathophysiology of inflammation. This transformation can be achieved by establishing robust links between inflammatory biomarkers and symptom‐specific subtypes of depression, tailoring anti‐inflammatory therapeutic interventions to high‐risk demographic groups, and advancing multidimensional mechanistic models that comprehensively elucidate the intricate interplay between inflammation and depression.

## Author Contributions

**Bei Hu**: data curation, writing – original draft. **Moshui Shan**: conceptualization, writing – review and editing. **Shuhua Wang**: data curation, writing – review and editing, formal analysis. **Yu Pan**: conceptualization, writing – review and editing. **Siyi Zhao**: writing – original draft.

## Funding

The authors have nothing to report.

## Conflicts of Interest

The authors declare no conflicts of interest.

## Supporting information




**Supplementary Table**: brb371541‐sup‐0001‐TableS1–S11.docx

## Data Availability

The datasets analyzed in the current study are available in the repository of the National Center for Health Statistics (NCHS), Centers for Disease Control and Prevention (CDC) (https://wwwn.cdc.gov/nchs/nhanes/Default.aspx).
