# Supplementary material for: Association Between Systemic Inflammation Response Index and Specific Depressive Symptoms: Evidence From a Cross‐Sectional Survey
Source: Brain Behav. 2026 Jun 8;16(6):e71541. doi: 10.1002/brb3.71541 (PMC13247137; doi:10.1002/brb3.71541)
Supplement: Supplementary file 1 — Supplementary Table: brb371541‐sup‐0001‐TableS1–S11.docx [file BRB3-16-e71541-s001.docx]

**Supplementary Table S1 Item descriptions of PHQ-9**

| Term | Item description |
| --- | --- |
| Anhedonia | Have little interest in doing things |
| Depressed mood | Feeling down, depressed, or hopeless |
| Sleep disturbance | Trouble sleeping or sleeping too much |
| Fatigue | Feeling tired or having little energy |
| Appetite change | Poor appetite or overeating |
| Low self-esteem | Feeling bad about yourself |
| Concentration problems | Trouble concentrating on things |
| Psychomotor changes | Moving or speaking slowly or too fast |
| Suicidal ideation | Thought you would be better off dead |

**Supplementary Table S2 The detailed descriptions of medical conditions (Weighted)**

| Reported disease | No. (%) of Participants |
| --- | --- |
| Asthma | 2502(8.14) |
| Anemia | 1161(3.19) |
| Arthritis | 6971(22.03) |
| Liver Condition | 576(1.76) |
| Thyroid Problem | 1946(7.26) |
| Cardiovascular disease | 2234(5.83) |
| Hypertension | 13738(41.74) |
| Diabetes | 4630(11.30) |
| Dyslipidemia | 7975(25.53) |

**Supplementary Table S3 multivariable logistic regression analyses for SIRI and specific depressive symptom (Asthma excluded) ***

|  | OR (95%CI) | p value |
| --- | --- | --- |
| Anhedonia | 1.13(1.03,1.23) | 0.008 |
| Depressed mood | 1.07(0.97,1.19) | 0.17 |
| Sleep disturbance | 1.10(1.03,1.18) | 0.005 |
| Fatigue | 1.12(1.04,1.20) | 0.003 |
| Appetite change | 1.10(1.01,1.20) | 0.03 |
| Low self-esteem | 1.03(0.92,1.15) | 0.63 |
| Concentration problems | 1.06(0.96,1.17) | 0.26 |
| Psychomotor changes | 1.05(0.90,1.21) | 0.53 |
| Suicidal ideation | 0.95(0.76,1.20) | 0.68 |

Abbreviation: odds ratio (OR), confidence interval (CI), systemic inflammation response index (SIRI).

*****Adjusted for age, sex, race, education level, marital status, poverty income ratio, body mass index, medical conditions (anemia, arthritis, liver conditions, thyroid problems, cardiovascular disease, hypertension, diabetes, and dyslipidemia), smoking status, alcohol use and antidepressant use.

**Supplementary Table S4 multivariable logistic regression analyses for SIRI and specific depressive symptom (Anemia excluded)**

|  | OR (95%CI) | p value |
| --- | --- | --- |
| Anhedonia | 1.13(1.04,1.22) | 0.005 |
| Depressed mood | 1.08(0.97,1.19) | 0.16 |
| Sleep disturbance | 1.12(1.05,1.19) | 0.001 |
| Fatigue | 1.11(1.04,1.20) | 0.003 |
| Appetite change | 1.11(1.02,1.21) | 0.02 |
| Low self-esteem | 1.04(0.93,1.16) | 0.50 |
| Concentration problems | 1.06(0.97,1.17) | 0.18 |
| Psychomotor changes | 1.03(0.89,1.18) | 0.72 |
| Suicidal ideation | 0.94(0.76,1.18) | 0.61 |

Abbreviation: odds ratio (OR), confidence interval (CI), systemic inflammation response index (SIRI).

*****Adjusted for age, sex, race, education level, marital status, poverty income ratio, body mass index, medical conditions (asthma, arthritis, liver conditions, thyroid problems, cardiovascular disease, hypertension, diabetes, and dyslipidemia), smoking status, alcohol use and antidepressant use.

**Supplementary Table S5 multivariable logistic regression analyses for SIRI and specific depressive symptom (Arthritis excluded)**

|  | OR (95%CI) | p value |
| --- | --- | --- |
| Anhedonia | 1.10(1.00,1.21) | 0.04 |
| Depressed mood | 1.07(0.95,1.21) | 0.24 |
| Sleep disturbance | 1.13(1.05,1.22) | 0.001 |
| Fatigue | 1.11(1.03,1.19) | 0.006 |
| Appetite change | 1.10(1.00,1.21) | 0.04 |
| Low self-esteem | 1.02(0.91,1.13) | 0.78 |
| Concentration problems | 1.08(0.97,1.20) | 0.16 |
| Psychomotor changes | 1.00(0.86,1.15) | 0.95 |
| Suicidal ideation | 0.95(0.74,1.22) | 0.70 |

Abbreviation: odds ratio (OR), confidence interval (CI), systemic inflammation response index (SIRI).

*****Adjusted for age, sex, race, education level, marital status, poverty income ratio, body mass index, medical conditions (asthma, anemia, liver conditions, thyroid problems, cardiovascular disease, hypertension, diabetes, and dyslipidemia), smoking status, alcohol use and antidepressant use.

**Supplementary Table S6 multivariable logistic regression analyses for SIRI and specific depressive symptom (Liver condition excluded)**

|  | OR (95%CI) | p value |
| --- | --- | --- |
| Anhedonia | 1.12(1.03,1.21) | 0.008 |
| Depressed mood | 1.07(0.97,1.18) | 0.16 |
| Sleep disturbance | 1.12(1.04,1.19) | 0.001 |
| Fatigue | 1.11(1.04,1.20) | 0.003 |
| Appetite change | 1.10(1.01,1.19) | 0.03 |
| Low self-esteem | 1.03(0.92,1.15) | 0.59 |
| Concentration problems | 1.06(0.97,1.16) | 0.19 |
| Psychomotor changes | 1.01(0.88,1.16) | 0.83 |
| Suicidal ideation | 0.96(0.78,1.19) | 0.74 |

Abbreviation: odds ratio (OR), confidence interval (CI), systemic inflammation response index (SIRI).

*****Adjusted for age, sex, race, education level, marital status, poverty income ratio, body mass index, medical conditions (asthma, anemia, arthritis, thyroid problems, cardiovascular disease, hypertension, diabetes, and dyslipidemia), smoking status, alcohol use and antidepressant use.

**Supplementary Table S7 multivariable logistic regression analyses for SIRI and specific depressive symptom (Thyroid problem excluded)**

|  | OR (95%CI) | p value |
| --- | --- | --- |
| Anhedonia | 1.14(1.05,1.24) | 0.002 |
| Depressed mood | 1.08(0.98,1.19) | 0.12 |
| Sleep disturbance | 1.11(1.04,1.19) | 0.002 |
| Fatigue | 1.11(1.04,1.19) | 0.003 |
| Appetite change | 1.12(1.04,1.21) | 0.006 |
| Low self-esteem | 1.04(0.93,1.16) | 0.47 |
| Concentration problems | 1.05(0.96,1.16) | 0.29 |
| Psychomotor changes | 0.99(0.86,1.15) | 0.93 |
| Suicidal ideation | 0.90(0.72,1.13) | 0.37 |

Abbreviation: odds ratio (OR), confidence interval (CI), systemic inflammation response index (SIRI).

*****Adjusted for age, sex, race, education level, marital status, poverty income ratio, body mass index, medical conditions (asthma, anemia, arthritis, liver conditions, cardiovascular disease, hypertension, diabetes, and dyslipidemia), smoking status, alcohol use and antidepressant use.

**Supplementary Table S8 multivariable logistic regression analyses for SIRI and specific depressive symptom (Hypertension excluded)**

|  | OR (95%CI) | p value |
| --- | --- | --- |
| Anhedonia | 1.17(1.02,1.33) | 0.02 |
| Depressed mood | 1.12(0.98,1.27) | 0.10 |
| Sleep disturbance | 1.17(1.08,1.28) | <0.001 |
| Fatigue | 1.13(1.03,1.23) | 0.01 |
| Appetite change | 1.12(1.00,1.25) | 0.05 |
| Low self-esteem | 1.04(0.89,1.22) | 0.61 |
| Concentration problems | 1.10(0.98,1.23) | 0.11 |
| Psychomotor changes | 1.00(0.81,1.24) | 0.98 |
| Suicidal ideation | 1.01(0.78,1.31) | 0.96 |

Abbreviation: odds ratio (OR), confidence interval (CI), systemic inflammation response index (SIRI).

*****Adjusted for age, sex, race, education level, marital status, poverty income ratio, body mass index, medical conditions (asthma, anemia, arthritis, liver conditions, thyroid problems, cardiovascular disease, diabetes, and dyslipidemia), smoking status, alcohol use and antidepressant use.

**Supplementary Table S9 multivariable logistic regression analyses for SIRI and specific depressive symptom (Cardiovascular disease excluded)**

|  | OR (95%CI) | p value |
| --- | --- | --- |
| Anhedonia | 1.14(1.05,1.24) | 0.003 |
| Depressed mood | 1.08(0.98,1.19) | 0.14 |
| Sleep disturbance | 1.12(1.04,1.19) | 0.002 |
| Fatigue | 1.13(1.06,1.21) | <0.001 |
| Appetite change | 1.11(1.02,1.21) | 0.02 |
| Low self-esteem | 1.04(0.94,1.16) | 0.46 |
| Concentration problems | 1.08(0.99,1.18) | 0.08 |
| Psychomotor changes | 1.02(0.88,1.18) | 0.76 |
| Suicidal ideation | 0.95(0.77,1.19) | 0.67 |

Abbreviation: odds ratio (OR), confidence interval (CI), systemic inflammation response index (SIRI).

*****Adjusted for age, sex, race, education level, marital status, poverty income ratio, body mass index, medical conditions (asthma, anemia, arthritis, liver conditions, thyroid problems, hypertension, diabetes, and dyslipidemia), smoking status, alcohol use and antidepressant use.

**Supplementary Table S10 multivariable logistic regression analyses for SIRI and specific depressive symptom (Dyslipidemia excluded)**

|  | OR (95%CI) | p value |
| --- | --- | --- |
| Anhedonia | 1.21(1.01,1.44) | 0.04 |
| Depressed mood | 1.07(0.95,1.21) | 0.25 |
| Sleep disturbance | 1.12(1.03,1.21) | 0.007 |
| Fatigue | 1.11(1.02,1.20) | 0.02 |
| Appetite change | 1.12(1.00,1.26) | 0.05 |
| Low self-esteem | 1.05(0.93,1.18) | 0.46 |
| Concentration problems | 1.05(0.95,1.16) | 0.32 |
| Psychomotor changes | 1.08(0.93,1.26) | 0.33 |
| Suicidal ideation | 0.91(0.70,1.18) | 0.47 |

Abbreviation: odds ratio (OR), confidence interval (CI), systemic inflammation response index (SIRI).

*****Adjusted for age, sex, race, education level, marital status, poverty income ratio, body mass index, medical conditions (asthma, anemia, arthritis, liver conditions, thyroid problems, cardiovascular disease, hypertension, and diabetes), smoking status, alcohol use and antidepressant use.

**Supplementary Table S11 multivariable logistic regression analyses for SIRI and specific depressive symptom (Diabetes excluded)**

|  | OR (95%CI) | p value |
| --- | --- | --- |
| Anhedonia | 1.13(1.02,1.24) | 0.02 |
| Depressed mood | 1.11(0.99,1.24) | 0.06 |
| Sleep disturbance | 1.12(1.04,1.21) | 0.002 |
| Fatigue | 1.10(1.02,1.18) | 0.01 |
| Appetite change | 1.12(1.03,1.22) | 0.01 |
| Low self-esteem | 1.07(0.95,1.19) | 0.26 |
| Concentration problems | 1.05(0.96,1.15) | 0.32 |
| Psychomotor changes | 1.00(0.85,1.18) | 0.98 |
| Suicidal ideation | 0.93(0.74,1.17) | 0.55 |

Abbreviation: odds ratio (OR), confidence interval (CI), systemic inflammation response index (SIRI).

*****Adjusted for age, sex, race, education level, marital status, poverty income ratio, body mass index, medical conditions (asthma, anemia, arthritis, liver conditions, thyroid problems, cardiovascular disease, hypertension, and dyslipidemia), smoking status, alcohol use and antidepressant use.
